# Erythrocyte levels of n-3 polyunsaturated fatty acids and incidence of frailty after a 6-year follow-up: the Korean frailty and aging cohort study

**DOI:** 10.3389/fnut.2025.1569832

**Published:** 2025-07-16

**Authors:** Jueun Kim, Miji Kim, Chang Won Won, Yongsoon Park

**Affiliations:** ^1^Department of Food and Nutrition, Hanyang University, Seoul, Republic of Korea; ^2^Department of Health Sciences and Technology, College of Medicine, Kyung Hee University, Seoul, Republic of Korea; ^3^Department of Family Medicine, College of Medicine, Kyung Hee University, Seoul, Republic of Korea

**Keywords:** community-dwelling older adults, prospective cohort study, incidence of frailty, biomarker of n-3 polyunsaturated fatty acids, omega-3 index

## Abstract

Previous studies have shown that the blood levels of n-3 polyunsaturated fatty acids (PUFA) are inversely associated with the prevalence of frailty, but associations with frailty incidence remain unknown. We examined the hypothesis that the erythrocyte levels of n-3 PUFA such as eicosapentaenoic acid (EPA) and docosahexaenoic acid (DHA) are inversely associated with the incidence of frailty after a 6-year follow-up. Using the Korean Frailty and Aging Cohort Study data, 1,119 community-dwelling Korean participants aged 70–84 years without frailty were observed for 6 years. Frailty was defined using the Cardiovascular Health Study index. In the multivariable adjusted model, the incidence of frailty was 11.1% after a 6-year follow-up and inversely associated with the Omega-3 Index (sum of EPA + DHA) (HR: 0.47; 95% CI: 0.27–0.84; *P* for trend = 0.005), and DHA levels (HR: 0.36; 95% CI: 0.19–0.68; *P* for trend = 0.003). Regarding frailty components, the incidence of low physical activity, slow walking speed, and weight loss were inversely associated with the Omega-3 Index and DHA levels. The Omega-3 Index (*p* = 0.043) and DHA levels (*p* = 0.019) differed significantly among the frailty transition groups (persistence, reversal, and deterioration). All-cause mortality was inversely associated with the Omega-3 Index (*p* = 0.011), and EPA (*p* = 0.012) and DHA levels (*p* = 0.032). The incidence of frailty was inversely associated with the Omega-3 Index and erythrocyte DHA levels, suggesting that interventions with n-3 PUFA are beneficial for preventing the progression of frailty and mortality among community-dwelling older adults in Korea.

## 1 Introduction

Frailty is characterized by unintentional weight loss, exhaustion, low grip strength, slow walking speed, and low physical activity, which lead to increased vulnerability associated with disability, hospitalization, and mortality (1). In the light of population aging, frailty has become an important condition, with an estimated global incidence of 13.6% (2).

Although the pathophysiological mechanisms that lead to and underlie the progression of frailty are not fully understood, n-3 polyunsaturated fatty acids (PUFA), which are abundant in fish, are of particular interest, given their well-known anti-inflammatory role and the contribution of inflammation to the aging process (3). The consumption of fish and oily fish has been associated with lower odds of frailty prevalence among Korean older adults from the Korean Frailty and Aging Cohort Study (KFACS) (4), Japanese women (5, 6), Irish older adults (7), British older adults from UK Biobank (8), and Brazilian adults (9). In addition, the prevalence of frailty is inversely associated with n-3 PUFA intake in American adults (10), and supplementation with fish oil in British older adults from UK Biobank (8). Our previous studies reported that the blood levels of the n-3 PUFA, eicosapentaenoic acid (EPA; 20:5n3), and docosahexaenoic acid (DHA; 22:6n3) are inversely associated with the prevalence of frailty in Korean older adults from KFACS (11) and British older adults from UK Biobank (8).

Furthermore, fish consumption reduced the incidence of frailty in Chinese older adults during a 3-year follow-up (12) and in Spanish adults from the ENRICA cohort during a 3.5-year follow-up (13). The incidence of pre-frailty was also inversely associated with fish intake in Norwegian adults during an 8-year follow-up (14). A meta-analysis of clinical trials showed that supplementation with EPA and DHA improved musculoskeletal health in older participants (15, 16). N-3 PUFA are known to improve muscle function, strength, and mass, as they reduce the breakdown of muscle protein by suppressing inflammatory cytokines, stimulating muscle protein synthesis via the mammalian target of rapamycin (mTOR) signaling pathway, and modulating neurotransmission in animal models (17).

To the best of our knowledge, no study has evaluated the association between frailty incidence and blood levels of n-3 PUFA, an objective biomarker for dietary fish intake (18). The aim of the present study was to examine the hypothesis that erythrocyte levels of EPA and DHA, and Omega-3 Index (sum of EPA + DHA) are inversely associated with the incidence of frailty after a 6-year follow-up in Korean community-dwelling older adults.

## 2 Methods

### 2.1 Participant selection

**Figure 1:**
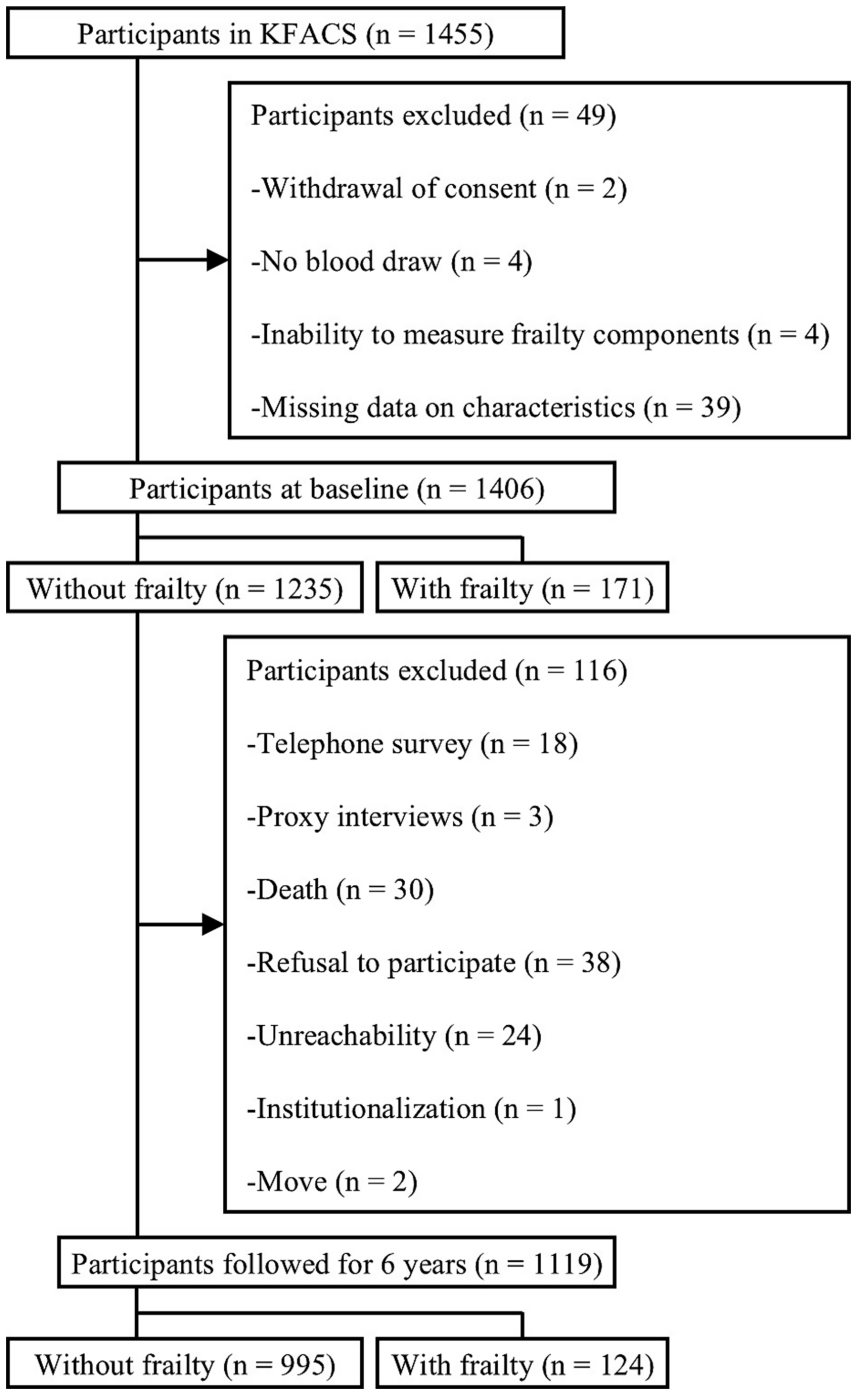
Flowchart of the process of participant selection for the 6-year follow-up. KFACS, Korean frailty and aging cohort study.

KFACS is an ongoing multicenter longitudinal cohort study of community-dwelling adults aged 70–84 years (19). Participants stratified by age and sex were recruited from eight hospitals and two public health centers in urban and rural areas of Korea. Of the total 1,455 participants recruited in KFACS in 2017, 49 were excluded because of withdrawal of consent (*n* = 2), no blood draw (*n* = 4), inability to measure frailty components (*n* = 4), and missing data on characteristics (*n* = 39) (Figure 1). Among the 1,406 participants included in the present study at baseline of 2017, 1,235 non-frail participants were followed up in 2019, 2021, and 2023. Thus, during 6-year follow-up period, frailty and mortality were measured every 2 years. For the final analysis of frailty incidence, 1,119 participants were included after excluding those without frailty measurements owing to telephone surveys (*n* = 18), proxy interviews (*n* = 3), death (*n* = 30), refusal to participate (*n* = 38), unreachability (*n* = 24), institutionalization (*n* = 1), and moving out (*n* = 2). Regarding the all-cause mortality analysis, 1,367 participants with and without frailty were observed for a follow-up period of 6-years, after excluding 39 participants with unknown dates of death. To determine transitions in frailty status, 1,107 participants without frailty at baseline were observed for a follow-up period of 6 years, after excluding 12 participants for whom pre-frailty could not be assessed. The time-to-event variable was defined as the time from the date of the baseline evaluation to the end of the follow-up period or mortality. The KFACS protocol was approved by the Institutional Review Boards (KHUH-2015-12-103-107, 2021–05–081-039, and HYUIRB-202407-025), and written informed consent was obtained from all participants.

### 2.2 Frailty assessment and transitions

The Cardiovascular Health Study (CHS) frailty index assesses unintentional weight loss, exhaustion, low handgrip strength, slow walking speed, and low physical activity (1). Participants with three or more components were defined with frailty, otherwise without. Those without signs of frailty were further classified as robust if they met none and with pre-frailty if they met one or two of the components. Unintentional weight loss was determined as a loss of ≥ 4.5 kg or 5% of body weight in the previous year. Exhaustion was evaluated using two questions from the Center for Epidemiological Studies Depression (CES-D) scale, and defined as a positive response to either one of the questions, “I felt that everything I did was an effort” or “I could not get going” for three or more days per week (19). Low physical activity was assessed using the International Physical Activity Questionnaire, with criteria set at ≤ 494.65 kcal/week for men and ≤ 283.50 kcal/week for women (19). Walking speed was assessed by walking 4 m, with 1.5 m before and after the walkway taken into consideration for acceleration and deceleration, and slow walking speed was defined as < 1 m/s (20). Hand grip strength was assessed on two separate occasions for each hand using a digital hand grip dynamometer (TKK-5401; Takei Scientific Instruments CO, Ltd., Tokyo, Japan), and low hand grip strength was categorized as maximal grip strength < 28 kg for men and < 18 kg for women (20). Transitions regarding frailty were divided into three groups according to changes in status from baseline to the 6-year follow-up: persistence (persistence of the robust or pre-frail status), reversal (from pre-frail to robust status), and deterioration (from robust to pre-frail or frail, or from pre-frail to frail status).

### 2.3 Measurement of erythrocyte fatty acid composition

At the baseline visit in 2017, fasting blood samples were obtained, and the fatty acid composition of the erythrocytes was analyzed as previously described (21). Briefly, the isolated erythrocytes were methylated by the addition of boron trifluoride methanol-benzene (Sigma-Aldrich, St. Louis, MO, US) for 10 min at 100°C. Fatty acid methyl esters were extracted with hexane and analyzed using gas chromatography (Shimadzu 2010AF; Shimadzu Scientific Instrument, Kyoto, Japan) with a 0.20 μm film capillary column (SP2560; Supelco, Bellefonte, PA, US). The contents of 27 fatty acids within the erythrocytes were identified via comparisons with known standards (GLC-727; Nu-Check Prep, Elysian, MN, US), and expressed as percentages of the total identified fatty acids. The coefficient of variation for a quality control sample was 4.4%.

### 2.4 Covariates

Information regarding age, sex, education, economic, smoking, living, and disease status, sleep duration and history of falls in the previous year was collected using the standardized questionnaire by the trained interviewers. Weight was measured to the nearest 0.1 kg using a portable digital scale, and height was measured to the nearest 0.1 cm using a measuring tape. Body mass index (BMI) was calculated as weight divided by height squared, and smoking status was classified as either current smoker or non-smoker. A low economic status was identified as recipients of the National Basic Livelihood Security system or Medical Beneficial system (19). Comorbid status was determined by the number of the following diseases: hypertension, myocardial infarction, heart failure, angina, cerebral ischemia, arthritis, asthma, chronic obstructive pulmonary disease, diabetes mellitus, renal disease, or cancer. Polypharmacy was indicated by the use of five or more prescribed drugs, and impaired cognitive function was defined as a Mini-Mental State Examination (MMSE) score < 24 (19). Nutritional status was evaluated using the Korean version of the short form of the Mini Nutritional Assessment (MNA); a score of 12–14 indicated normal nutritional status; 8–11 was associated with the risk of malnutrition; and a score < 7 indicated malnutrition (19). Sleep duration was classified into three categories: < 7 h, 7–8 h, and > 8 h, based on the recommendations for sleep duration for older adults by the National Sleep Foundation (22).

### 2.5 Statistical analysis

Statistical analyses were performed using the SPSS software (version 27.0; SPSS Inc., Chicago, IL, US). The Kolmogorov–Smirnov test was used to evaluate the normal distribution of the variables. The baseline characteristics of the participants are expressed as the mean ± standard deviation (SD) for continuous variables, and as the number of participants (percentage distribution) for categorical variables. Differences between groups were assessed using the independent *t*-test or Mann–Whitney *U*-test for continuous variables and a chi-square test for categorical variables. To minimize potential bias in the multivariate models, covariates with a *p* < 0.20 such as age, sex, comorbidity, polypharmacy, cognitive impairment, and nutritional status were selected as confounding factors, and were included in the fully adjusted model. The erythrocyte levels of fatty acids were compared between participants with and without frailty, and among the frailty transition groups using analysis of covariance (ANCOVA) after adjusting for the covariates, followed by the Dunn-Bonferroni *post-hoc* test. The association between frailty incidence or all-cause mortality and erythrocyte levels of fatty acids was expressed as HRs and 95% CIs using COX proportional hazards regression analysis after adjusting for covariates. The lowest quintile of the erythrocyte fatty acid levels was considered the reference group, and *P* for trend was calculated using the median value of each quintile.

## 3 Results

The incidence of frailty was 11.1% (*n* = 124) after a 6-year follow-up. Frailty was associated with older age, less education, more comorbidities, cognitive impairment, female sex, living alone, and polypharmacy (Table 1). There were no significant differences between participants with and without frailty regarding BMI, smoking, economic, or nutritional status, episodes of falls, or sleep duration.

**Table 1:** Baseline characteristics of participants with and without frailty after a 6-year follow-up.

Variables	Non-frailty (*n* = 995)	Frailty (*n* = 124)	*p*-value
Age, years	75.13 ± 3.60	77.39 ± 3.84	<0.001
Women, *n* (%)	497 (49.9)	79 (63.7)	0.004
Body mass index, kg/m^2^	24.53 ± 2.88	24.60 ± 3.43	0.918
Current smoker, *n* (%)	53 (5.3)	7 (5.6)	0.882
Education years, *n* (%)			<0.001
≥ 7	665 (66.8)	56 (45.2)	
0–6	330 (33.2)	68 (54.8)	
Low economic status, *n* (%)	63 (6.3)	10 (8.1)	0.461
Living alone, *n* (%)	176 (17.7)	32 (25.8)	0.028
Comorbidities, *n* (%)			<0.001
< 2	658 (66.1)	56 (45.2)	
≥ 2	337 (33.9)	68 (54.8)	
Polypharmacy, *n* (%)	269 (27.0)	54 (43.5)	<0.001
Cognitive impairment, *n* (%)	124 (12.5)	42 (33.9)	<0.001
Nutritional status, *n* (%)			0.072
Normal	869 (87.3)	100 (80.6)	
At risk of malnutrition	123 (12.4)	23 (18.5)	
Malnutrition	3 (0.3)	1 (0.8)	
Falls in the past year, *n* (%)	170 (17.1)	30 (24.2)	0.051
Sleep duration, *n* (%)			
< 7 h	642 (64.5)	77 (62.1)	0.863
7–8 h	306 (30.8)	41 (33.1)	
> 8 h	47 (4.7)	6 (4.8)	

The data are presented as the mean ± SD or the number of participants (percentage distribution), as appropriate. *p*-values were analyzed using the independent *t*-test for parametric continuous variables, the Mann–Whitney *U* test for non-parametric continuous variables, and the chi-squared test for categorical variables.

The participants with frailty had a lower Omega-3 Index and erythrocyte levels of DHA but higher erythrocyte levels of *α*-linolenic acid (ALA; 18:3n3) than those without (Table 2). In the multivariable adjusted model, the incidence of frailty was inversely associated with the Omega-3 Index and DHA level (Table 3). The incidence of frailty was significantly lower in participants in the fourth and the highest quintiles of the Omega-3 Index, and in those in the highest DHA quintile than in those in the lowest quintiles.

**Table 2:** Baseline erythrocyte fatty acid composition of participants with and without frailty after a 6-year follow-up.

%	Non-frailty (*n* = 995)	Frailty (*n* = 124)	*P-*value
14:0	0.34 ± 0.09	0.35 ± 0.10	0.928
16:0	21.91 ± 0.98	22.03 ± 1.11	0.618
18:0	17.11 ± 0.73	17.11 ± 0.78	0.929
16:1n7	0.43 ± 0.20	0.49 ± 0.23	0.116
18:1n9	13.80 ± 0.95	13.90 ± 0.95	0.562
18:3n3	0.36 ± 0.23	0.43 ± 0.33	0.010
20:5n3	2.25 ± 0.87	2.14 ± 0.89	0.261
22:5n3	3.40 ± 0.50	3.47 ± 0.52	0.462
22:6n3	9.62 ± 1.44	9.19 ± 1.53	0.007
Omega-3 Index	11.87 ± 2.05	11.33 ± 2.04	0.016
18:2n6	9.79 ± 1.62	9.52 ± 1.62	0.738
20:3n6	1.54 ± 0.29	1.59 ± 0.29	0.252
20:4n6	13.70 ± 1.45	13.99 ± 1.55	0.401
22:4n6	2.09 ± 0.48	2.16 ± 0.55	0.596
16:1n7t	0.12 ± 0.09	0.12 ± 0.04	0.698
18:1t	0.31 ± 0.08	0.30 ± 0.07	0.172
18:2n6t	0.17 ± 0.05	0.17 ± 0.05	0.628

The data are presented as the mean ± SD. *P*-value was determined using ANCOVA after adjustment for age, sex, comorbidity, polypharmacy, cognitive impairment, and nutritional status.

**Table 3:** Cox proportional hazards regression analysis of erythrocyte n-3 polyunsaturated fatty acid levels for frailty incidence after a 6-year follow-up.

	Quintiles of erythrocyte fatty acid levels					*P* for trend	Fatty acid levels, continuous^a^	
	Q1	Q2	Q3	Q4	Q5		HR (95% CI)	*P-*value
18:3n3								
Non-frailty/frailty, *n*	199/25	206/18	198/25	203/21	189/35			
Cutoff, %	≤ 0.20	0.20 < to ≤ 0.26	0.26 < to ≤ 0.34	0.34 < to ≤ 0.50	> 0.50			
HR (95% CI)	1.00	0.77 (0.42–1.43)	1.17 (0.66–2.05)	1.03 (0.57–1.87)	1.47 (0.86–2.49)	0.054	2.95 (1.62–5.35)	< 0.001
20:5n3								
Non-frailty/frailty, *n*	197/27	189/35	204/19	204/20	201/23			
Cutoff, %	≤1.50	1.50 < to ≤ 1.92	1.92 < to ≤ 2.33	2.33 < to ≤ 2.88	> 2.88			
HR (95% CI)	1.00	1.22 (0.74–2.04)	0.63 (0.35–1.14)	0.95 (0.53–1.71)	0.78 (0.45–1.37)	0.232	0.86 (0.70–1.05)	0.143
22:5n3								
Non-frailty/frailty, *n*	204/20	197/27	201/22	198/26	195/29			
Cutoff, %	≤2.97	2.97 < to ≤ 3.27	3.27 < to ≤ 3.54	3.54 < to ≤ 3.81	> 3.81			
HR (95% CI)	1.00	1.33 (0.74–2.38)	1.16 (0.63–2.13)	1.27 (0.70–2.29)	1.24 (0.69–2.21)	0.593	1.18 (0.83–1.67)	0.356
22:6n3								
Non-frailty/frailty, *n*	193/31	195/29	198/25	200/24	209/15			
Cutoff, %	≤8.36	8.36 < to ≤ 9.25	9.25 < to ≤ 9.90	9.90 < to ≤ 10.79	>10.79			
HR (95% CI)	1.00	1.01 (0.61–1.68)	0.85 (0.50–1.45)	0.91 (0.53–1.58)	0.36 (0.19–0.68)	0.003	0.81 (0.72–0.91)	< 0.001
Omega-3 Index								
Non-frailty/frailty, *n*	190/34	200/24	193/30	207/17	205/19			
Cutoff, %	≤10.02	10.02 < to ≤ 11.25	11.25 < to ≤ 12.26	12.26 < to ≤ 13.46	>13.46			
HR (95% CI)	1.00	0.72 (0.43–1.22)	0.95 (0.58–1.56)	0.49 (0.27–0.89)	0.47 (0.27–0.84)	0.005	0.87 (0.80–0.95)	0.002

Values are hazard ratios (HRs) with 95% confidence intervals (CIs) after adjusting for age, sex, comorbidity, polypharmacy, cognitive impairment, and nutritional status. *P* for trend was estimated using linear trend tests based on linear scores derived from the median erythrocyte levels of fatty acids in each quintile. ^a^Each point corresponds to a 1-unit increment in the erythrocyte levels of fatty acids.

**Figure 2:**
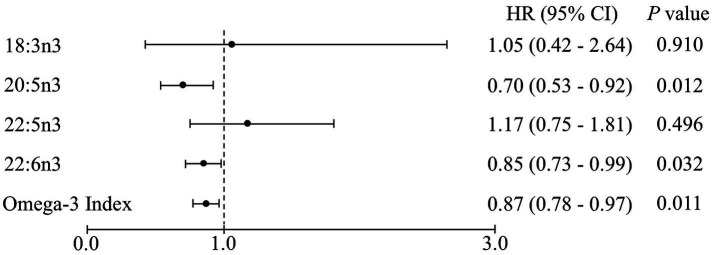
Cox proportional hazards regression analysis of the erythrocyte n-3 polyunsaturated fatty acid levels for all-cause mortality after a 6-year follow-up. Values are hazard ratios (HRs) with 95% confidence intervals (CIs) after adjusting for age, sex, body mass index, economic status, polypharmacy, and cognitive impairment. Each point corresponds to a 1-unit increment in the erythrocyte levels of fatty acids.

The Omega-3 Index and DHA levels were significantly different among the three frailty transition groups (persistence, reversal, and deterioration) but the differences did not remain in the *post-hoc* analyses (Table 4). Similar to the results regarding frailty transitions, all-cause mortality was 5.6% and inversely associated with the Omega-3 Index and EPA and DHA levels after 6 years in the multivariable adjusted model (Figure 2). The Omega-3 Index and DHA levels were inversely associated with incidence of weight loss, low physical activity, and slow walking speed (Table 5). The level of ALA was positively associated with incidence of weight loss, exhaustion, and low physical activity, and the level of docosapentaenoic acid (DPA; 22:5n3) was positively associated with the incidence of exhaustion.

**Table 4:** Erythrocyte levels of n-3 polyunsaturated fatty acids according to frailty transition after a 6-year follow-up.

%	Transition of frailty			*P*-value
	Persistence (*n* = 611)	Reversal (*n* = 144)	Deterioration (*n* = 352)	
18:3n3	0.36 ± 0.23	0.40 ± 0.25	0.38 ± 0.27	0.236
20:5n3	2.26 ± 0.87	2.18 ± 0.79	2.21 ± 0.91	0.467
22:5n3	3.39 ± 0.49	3.41 ± 0.52	3.43 ± 0.51	0.625
22:6n3	9.68 ± 1.41	9.37 ± 1.46	9.44 ± 1.51	0.019
Omega-3 index	11.95 ± 2.02	11.55 ± 1.96	11.65 ± 2.13	0.043

The data are presented as the mean ± SD. *p*-value was determined using ANCOVA followed by the Dunn-Bonferroni *post-hoc* test after adjusting for age, sex, comorbidity, polypharmacy, cognitive impairment, and nutritional status. Transitions in the status of frailty were divided into three groups according to changes from baseline to the 6-year follow-up: persistence (persistence of robust or pre-frail status), reversal (pre-frail to robust status), and deterioration (from robust to pre-frail or frail, or from pre-frail to frail status).

**Table 5:** Cox proportional hazards regression analysis of erythrocyte n-3 polyunsaturated fatty acid levels for each frailty component after a 6-year follow-up.

%	Weight loss (*n* = 169)		Exhaustion (*n* = 184)		Low PA (*n* = 83)		Slow WS (*n* = 198)		Low HGS (*n* = 168)	
	HR (95% CI)	*P-*value	HR (95% CI)	*P-*value	HR (95% CI)	*P-*value	HR (95% CI)	*P-*value	HR (95% CI)	*P-*value
18:3n3	2.10 (1.15–3.83)	0.016	2.23 (1.30–3.83)	0.004	2.74 (1.25–5.97)	0.011	1.10 (0.56–2.14)	0.786	1.51 (0.77–2.95)	0.233
20:5n3	0.95 (0.79–1.14)	0.603	1.09 (0.94–1.28)	0.262	0.89 (0.70–1.13)	0.325	0.90 (0.77–1.06)	0.205	0.93 (0.78–1.11)	0.399
22:5n3	0.98 (0.72–1.33)	0.896	1.45 (1.11–1.91)	0.007	1.16 (0.75–1.79)	0.495	1.01 (0.77–1.34)	0.935	0.97 (0.71–1.32)	0.835
22:6n3	0.85 (0.77–0.95)	0.003	0.94 (0.85–1.04)	0.252	0.84 (0.73–0.97)	0.019	0.89 (0.81–0.98)	0.016	0.92 (0.83–1.02)	0.128
Omega-3 index	0.91 (0.85–0.99)	0.021	0.99 (0.92–1.06)	0.760	0.90 (0.81–1.00)	0.040	0.93 (0.86–0.99)	0.025	0.95 (0.88–1.02)	0.156

Values are hazard ratios (HRs) with 95% confidence intervals (CIs) after adjusting for age, sex, comorbidity, polypharmacy, cognitive impairment, and nutritional status. Each point corresponds to a 1-unit increment in the erythrocyte levels of fatty acids. PA, physical activity; WS, walking speed; HGS, handgrip strength.

## 4 Discussion

The present study showed that the Omega-3 Index and erythrocyte levels of DHA were inversely associated with the incidence of frailty among community-dwelling Korean older adults aged 70–84 years after a 6-year follow-up. Our previous studies consistently reported that the blood levels of n-3 PUFA were inversely associated with lower odds for the prevalence of frailty in Korean older adults from KFACS (11) and British older adults from UK Biobank (8). Additionally, the risk of frailty prevalence was inversely associated with the intake of n-3 PUFA in American adults (10), and fish and oily fish consumption in Korean older adults from KFACS (4), Japanese older women (6), Japanese older women with rheumatoid arthritis (5), Irish older adults (7), British older adults from UK Biobank (8), and Brazilian adults (9). Our previous study also reported that the risk of frailty prevalence was inversely associated with fish oil supplementation in British older adults from UK Biobank (8). The frailty score was inversely correlated with intake of fish in Ecuadorian older adults (23) and fish oil in American adults (24). Furthermore, the blood levels of n-3 PUFA, consumption of n-3 PUFA and fish, and frequency of fish oil supplementation were lower in Korean older adults with frailty than in those without (4, 11), Chinese adults (25), Spanish older adults (26), American adults (10), British adults (27), Brazilian adults (9), and Taiwanese older adults (28).

Consistently, fish intake was inversely associated with the incidence of frailty in Chinese older adults during a 3-year follow-up (12) and the incidence of pre-frailty in Norwegian older adults during an 8-year follow-up (14). The ENRICA cohort studies also showed that the incidence of frailty was inversely associated with the intake of fish during a 3.5-year follow-up, but not associated with the intake of n-3 PUFA (13, 29). The blood levels of n-3 PUFA correlated with the dietary intake of n-3 PUFA, but n-3 PUFA intake might not be an objective biomarker for the status of n-3 PUFA in the body compared with the blood levels of n-3 PUFA (18). On the other hand, n-3 PUFA consumption is lower in older adults with frailty than in those without (29). The secondary analysis of the MAPT study reported that the erythrocyte levels of n-3 PUFA were not associated with the incidence of frailty (30). However, in that clinical trial of MAPT study, 49% of the participants were supplemented with 1 g/d of EPA + DHA, and their erythrocyte levels of n-3 PUFA increased from 5.9 to 9.3%, which could attenuate the association between n-3 PUFA content and incidence of frailty (31, 32). Three previous clinical trials showed that supplementation with 1 g/d of n-3 PUFA for 3–5 years had no significant impact on the incidence of frailty in older adults from America and Europe (33–35). In contrast, meta-analyses of clinical trials showed that n-3 PUFA supplementation improved muscle function and muscle strength in older adults (15, 16). Additionally, the intake of > 2 g/d of n-3 PUFA increased muscle mass in older adults (15), suggesting that higher doses of n-3 PUFA are needed to affect frailty status. N-3 PUFA are known to increase skeletal muscle mass and function; the mechanism involves inhibition of muscle protein breakdown by lowering the levels of inflammatory cytokines, enhanced muscle protein synthesis through protein kinase activation in the mTOR signaling pathway, and improving muscle strength by modulating neurotransmission in an animal model (17).

Regarding the transition between frailty states, the Omega-3 Index and erythrocyte levels of DHA were different among the persistence, reversal, and deterioration groups, suggesting that the n-3 PUFA blood levels had minimal effect on the prevention of frailty transition. On the other hand, n-3 PUFA intake in Japanese adults and fish intake in Korean older adults were not significantly different among the deterioration, persistence, and reversal groups (4, 36). The present study demonstrated transitions in frailty status among the robust or pre-frail groups at baseline, whereas the populations in other studies were Japanese adults with pre-frailty (36) and Korean older adults with robust, pre-frail, and frail status (4). Additionally, the incidence of frailty in the present study was 11.1%, higher than that in other studies—7% in Japanese adults (36) and 9.6% in Korean older adults (4)—and the sample size was also larger in the present study than in other studies. Frailty is known to be an independent predictor of mortality in older adults (1). In a meta-analysis of prospective cohort studies, the risk of all-cause mortality was significantly higher in older adults with frailty than in those without, suggesting that preventing frailty could reduce mortality (37). In fact, the Omega-3 Index and erythrocyte levels of DHA and EPA were inversely associated with all-cause mortality in the present study. Meta-analyses of prospective studies have consistently shown that the consumption of fish and n-3 PUFA, and the blood levels of EPA and DHA are inversely associated with all-cause mortality in Asian, American, Australian, and European adults (38, 39), suggesting that high n-3 PUFA levels can reduce mortality, partly by preventing the deterioration of frailty.

The present study showed that the incidences of slow walking speed, low physical activity, and weight loss were inversely associated with the Omega-3 Index and erythrocyte levels of DHA after a 6-year follow-up. Consistently, the prevalence of slow walking speed was inversely associated with the blood levels of n-3 PUFA in older Korean (11), British (8), and French adults (40), and in Italian adults (41). Prevalence of slow walking speed was also inversely associated with the intake of n-3 PUFA in older Finnish women (42) and with fish and oily fish intake and supplementation with fish oil in Korean (4) and British older adults (8). In a meta-analysis of clinical trials, the administration of n-3 PUFA, particularly ≥ 24 weeks, improved walking speed among older adults (15). Additionally, the prevalence of physical activity was positively associated with the blood levels of n-3 PUFA in Korean older adults (11), British older adults (8), and Japanese adults (43), and with oily fish intake and fish oil supplementation in British older adults (8). Consistent with the present study, the prevalence of weight loss was inversely associated with the serum levels of n-3 PUFA in Japanese adults (43). On the other hand, prevalence of weight loss was not associated with the erythrocyte levels of n-3 PUFA or fish intake in Korean older adults (4, 11) but positively associated with the intake of fish and n-3 PUFA in British older adults (8). A meta-analysis of clinical trials showed that supplementation with n-3 PUFA increased weight gain among participants with cancer with cachexia (44), while it had no effect on weight loss in participants with overweight and obesity (45), suggesting that effect of n-3 PUFA on weight changes varies depending on the body weight of the participants.

The erythrocyte levels of DHA, but not EPA, were significantly associated with the incidence of frailty after the 6-year follow-up in the present study. Supplementation with fish oil significantly increased the plasma concentrations of EPA-derived oxylipins, but not of DHA-derived oxylipins, in German adults (46). Kaur et al. (47) reported higher incorporation in skeletal muscles of isotopically labeled DHA after oral administration than of EPA, which is oxidized to a greater extent than DHA in rats, suggesting that EPA is not a good marker for dietary intake. In addition, DHA but not EPA effectively prevents palmitic acid–induced myosteatosis in skeletal muscle cells, resulting in improved muscle function (48).

In the present study, the incidence of frailty was positively associated with the erythrocyte levels of ALA, as a continuous variable only, and was not associated with DPA levels. The erythrocyte levels of ALA were inversely associated with appendicular lean mass in Belgian older adults (49). In contrast, ALA intake was not associated with the incidence of frailty in Spanish adults (29) or the frailty score in American adults (50). The ALA levels might not be a good biomarker for dietary intake because the erythrocyte level of ALA does not correlate with fish or n-3 PUFA intake (18). In addition, the erythrocyte levels and intake of DPA are not significantly different between Korean older adults from KFACS with and without frailty (11), and are not associated with the frailty score in American adults (50). The major dietary sources of DPA are meat and poultry, rather than fish or seafood (51).

The main strengths of the present study were firstly to show that the Omega-3 Index and erythrocyte levels of DHA are inversely associated with the incidence of frailty among older adults. However, this study had a few limitations. First, since Koreans have higher erythrocyte levels of n-3 PUFA than Western populations (52), the applicability of our results to diverse populations could be restricted. On the other hand, our previous studies reported that blood levels of n-3 PUFA was inversely associated with the prevalence of frailty among older adults both from KFACS and UK biobank, suggesting the generalizability of the results (8, 11). Second, despite controlling for confounding factors, there remains a potential for unobserved variables to have impacted the outcomes of the study, such as physical activity and C-reactive protein. Third, the power to detect all-cause mortality was low in the present study due to the small number of deaths.

## 5 Conclusion

The present study showed that the Omega-3 Index and erythrocyte levels of DHA were favorably related to the incidence of frailty, suggesting that n-3 PUFA are beneficial for preventing frailty and mortality. Future studies are necessary to confirm the dose-dependent effects of n-3 PUFA on the incidence of frailty in clinical trials, and mortality in large prospective cohorts.

## Data Availability

The data analyzed in this study is subject to the following licenses/restrictions: the data are not publicly available because of privacy and ethical restrictions. Requests to access these datasets should be directed to MK, mijiak@khu.ac.kr.
